# Invasive Pulmonary Aspergillosis in Patients with HBV-Related Acute on Chronic Liver Failure

**DOI:** 10.3390/jof10080571

**Published:** 2024-08-14

**Authors:** Man Yuan, Ning Han, Duoduo Lv, Wei Huang, Mengjie Zhou, Libo Yan, Hong Tang

**Affiliations:** 1 Center of Infectious Diseases, West China Hospital of Sichuan University, Chengdu 610041, China; yuanman@wchscu.cn (M.Y.); hn950917@foxmail.com (N.H.); lvduo1217@163.com (D.L.); 18009634236@163.com (W.H.); zmj_hx2023@foxmail.com (M.Z.); 2 Division of Infectious Diseases, State Key Laboratory of Biotherapy, Sichuan University, Chengdu 610041, China

**Keywords:** invasive pulmonary aspergillosis, voriconazole, acute on chronic liver failure, hepatitis B virus

## Abstract

Background: We aim to investigate the characteristics of invasive pulmonary aspergillosis (IPA) in patients with HBV-related acute on chronic liver failure (HBV-ACLF). Methods: A total of 44 patients with probable IPA were selected as the case group, and another 88 patients without lung infections were chosen as the control group. Results: HBV-ACLF patients with probable IPA had more significant 90-day mortality (38.6% vs. 15.9%, *p* = 0.0022) than those without. The white blood cell (WBC) count was the independent factor attributed to the IPA development [odds ratio (OR) 1.468, *p* = 0.027]. Respiratory failure was associated with the mortality of HBV-ACLF patients with IPA [OR 26, *p* = 0.000]. Twenty-seven patients received voriconazole or voriconazole plus as an antifungal treatment. Plasma voriconazole concentration measurements were performed as therapeutic drug monitoring in 55.6% (15/27) of the patients. The drug concentrations exceeded the safe range with a reduced dosage. Conclusions: The WBC count might be used to monitor patients’ progress with HBV-ACLF and IPA. The presence of IPA increases the 90-day mortality of HBV-ACLF patients mainly due to respiratory failure. An optimal voriconazole regimen is needed for such critical patients, and voriconazole should be assessed by closely monitoring blood levels.

## 1. Introduction

Invasive pulmonary aspergillosis (IPA) is a rapidly progressive, frequently fatal disease. It mainly occurs in immunocompromised patients, such as patients with neutropenia, patients with hematological malignancies, recipients of hematopoietic stem cell transplantation or solid organ transplantation, or patients undergoing corticosteroid treatment and other immunosuppressive therapies [1]. In recent years, studies have shown that IPA occurs in patients with decompensated cirrhosis and liver failure [2,3].

Liver failure is a life-threatening condition. Due to the rare survival of patients with liver failure, *Aspergillus* detection was limited; none of the genus *Aspergillus* was identified in patients with liver failure before 2006 [4]. Recently, with the advancement of diagnosis and treatment, the survival rate of patients with liver failure has been improved, and the detection rate of various fungal infections has also been significantly increased. Owing to multiple immunologic defects, patients with liver failure are prone to various infections, including bacterial, viral, and fungal infections such as IPA [4,5,6]. IPA lacks specific clinical manifestations in the early stage, and symptoms such as fever, cough, and dyspnea are easily overlooked by symptoms of liver failure or its complications.

Acute on chronic liver failure (ACLF) is a prevalent kind of liver failure as a result of chronic liver disease or an acute decompensation of an end-stage liver disease [7]. Hepatitis B virus (HBV) infection remains Asia’s leading cause of ACLF [7]. The studies for IPA in patients with HBV-related acute on chronic liver failure (HBV-ACLF) remain limited. In our retrospective study, we aim to investigate the prevalence, clinical manifestations, risk factors, outcomes, and antifungal treatment of IPA in patients with HBV-ACLF and to improve the prognosis of patients with HBV-ACLF complicated by IPA.

## 2. Materials and Methods

### 2.1. Study Subjects

This retrospective study included patients with probable IPA diagnosis admitted with ACLF to the West China Hospital of Sichuan University from February 2008 to September 2023. The data of patients without pulmonary infection were collected from the same databases. Clinical data from all selected patients were reviewed, including demographic features, predisposing factors, clinical manifestations, results of laboratory tests, chest computed tomography (CT) images, treatments, and prognosis. Informed consent was not obtained since it was a retrospective analysis of the collected data, and the participants’ identities were kept confidential. The study protocol was approved by the Ethics Committee of the West China Hospital of Sichuan University (Chengdu, Sichuan, China) following the ethical guidelines of the 1975 Declaration of Helsinki (approval number: 2023-1884). Subjects with other clinical liver diseases, such as autoimmune liver diseases, alcoholic liver disease, drug-induced liver injury, hepatocellular carcinoma, hematologic malignancy, bile duct obstruction, Wilson’s disease, chronic hepatitis C infection, or human immunodeficiency virus coinfection, were excluded.

### 2.2. Enrolment Criteria

ACLF was defined according to the following criteria specified by the Asian Pacific Association for the Study of the Liver [7] and the Guideline for Diagnosis and Treatment of Liver Failure in China [8]: (1) extreme fatigue with severe digestive symptoms such as apparent anorexia, abdominal distension, or nausea and vomiting; (2) the acute deterioration of pre-existing chronic liver disease/cirrhosis; (3) serum total bilirubin ≥ 178 μmol/L; and (4) coagulopathy (INR ≥ 1.5).

**Figure 1:**
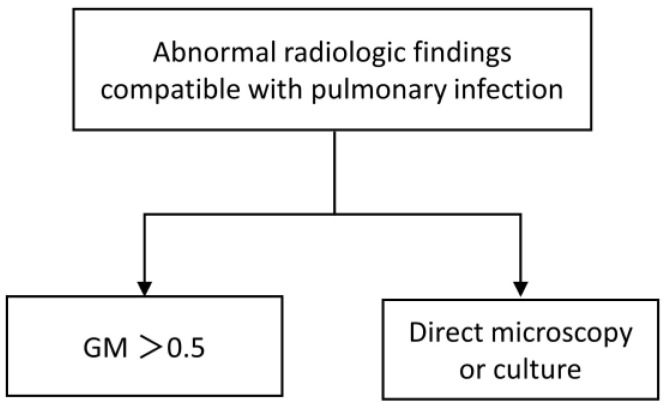
IPA criteria.

The diagnosis of IPA was classified as proven, probable, possible IPA, or *Aspergillus* colonization according to the definitions of the EORTC/MSG consensus [9]. Due to the severe coagulopathy and the concern of bleeding complications, none of the ACLF patients underwent bronchoscopy for needle aspiration biopsy, and only two patients underwent bronchoalveolar lavage. Considering ACLF as a host risk factor for IPA, patients with possible IPA were defined as having probable IPA if they met both of the following criteria: (1) the presence of abnormal radiologic findings compatible with pulmonary infection, including classical signs of IPA (dense, well-circumscribed lesions with or without a halo sign, air crescent sign, or cavity) or non-classical findings such as masses and (2) the presence of *Aspergillus* species indicated by direct test (direct microscopy or culture) or galactomannan (GM) antigen detected in plasma with a cut-off point value of 0.5 (Figure 1).

Patients with HBV-ACLF whose CT scans did not show infiltrates in the lung were included as controls. 

### 2.3. Patient Outcomes

The outcomes noted were the 90-day mortality.

### 2.4. Statistical Analyses

All statistical analyses were performed using SPSS version 22.0 (SPSS, Inc., Chicago, IL, USA). Continuous variables without a normal distribution are expressed as the median and inter-quartile range (IQR). Categorical variables are expressed as frequencies and percentages. The χ^2^ test or Fisher’s exact probability test was used to examine categorical variables such as sex and age. The Mann–Whitney U test was used to analyze continuous variables. Predictors for the development of IPA and mortality were determined by the odds ratio (OR) and 95% confidence interval (CI), which were calculated using multivariable binomial logistic regression analysis. Survival analysis was presented using the Kaplan–Meier method and compared by the log-rank test. A two-sided *p*-value < 0.05 was considered statistically significant.

## 3. Results

### 3.1. Study Populations and the Incidence of IPA

**Figure 2:**
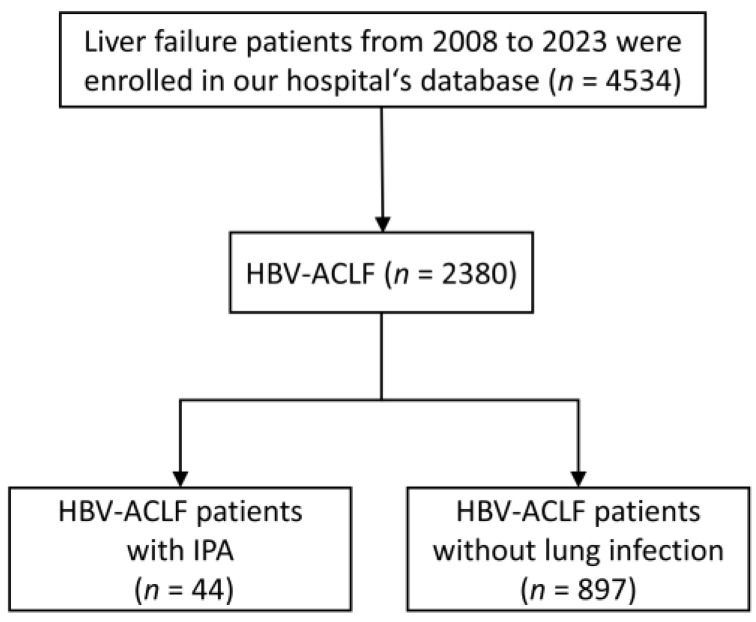
Flow chart of patient selection.

A diagram of the population selection in the study is shown in Figure 2. In this study, 4534 patients with liver failure were screened. Of these, the patients with HBV-ACLF were 2380, and with IPA were 44; the overall incidence of IPA in patients with HBV-ACLF was 44/2380 (1.84%). We enrolled 88 HBV-ACLF patients diagnosed with no lung infection by random sampling and compared their characteristics with those of 44 IPA patients.

**Figure 3:**
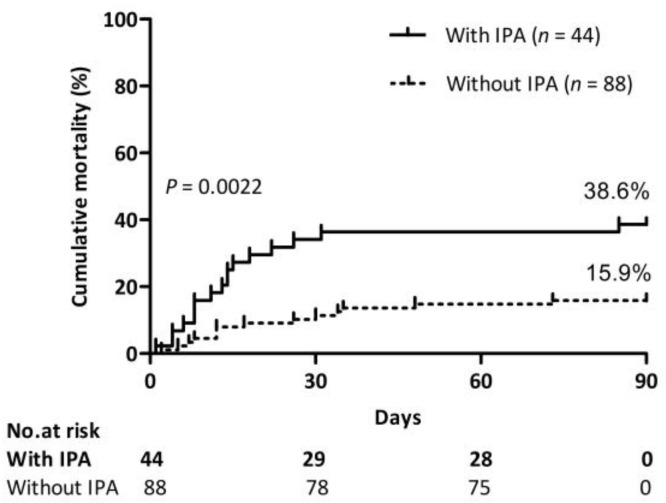
Impact of IPA on mortality. Cumulative 90-day mortality of patients with or without IPA. Log-rank test (38.6% vs. 15.9%, *p* = 0.0022).

Table 1 presents the differences in baseline characteristics, complications, laboratory findings, and outcome parameters between the IPA and non-IPA patients. The two groups did not differ in terms of age, sex, and hospital days. The percentage of underlying diseases of cardiovascular disease and diabetes mellitus type 2 was higher in the IPA group than in the non-IPA group. The incidence of upper gastrointestinal bleeding and septic shock in the IPA group was significantly higher than those in the non-IPA group. Compared to the non-IPA group, patients with IPA had lower ALT, AST, GLB, and Hb, a higher WBC count and neutrophil ratio, a lower platelet count, and higher procalcitonin. IPA developments were not attributed to the severity of liver failures, as indicated by MELD and MELD-Na score between these two groups. Antibiotic usage and steroid or immunosuppressant exposure were higher in the IPA group than in the non-IPA group. The 90-day mortality of patients with IPA was 38.6%, which was higher than that of patients without IPA (15.9%) (Figure 3).

### 3.2. Factors Associated with the Development of IPA

On multivariate logistic regression analysis, WBC count [odds ratio (OR) 1.468, *p* = 0.027] was the significant predictor for the development of IPA in patients with HBV-ACLF (Table 2). The parameter might have been involved in the development of IPA in patients with HBV-ACLF; therefore, measuring it might help monitor the occurrence of IPA.

### 3.3. Clinical Characteristics of Patients with IPA

Table 3 and Table 4 present the clinical characteristics of patients with IPA. Five patients were admitted to the intensive care unit. Mechanical ventilation (thirteen patients), artificial liver support system (thirty-one patients), liver transplantation (four patients), vasoactive agents (seven patients), and continuous renal replacement therapy (six patients) were applied. In addition, ten patients received steroid or immunosuppressant treatment. All patients with IPA used one or more antibiotics.

Patients with IPA had fever and respiratory symptoms, including cough, hemoptysis, and dyspnea. These were not specific symptoms but may have been useful indicators of the early stage of IPA. Imaging findings were significant for IPA diagnosis. Halo signs or air-crescent signs help diagnose IPA and could guide antifungal therapy at an early stage. In the study, the imaging results from the chest CT scan were not specific, and nodules were the most common change rather than the halo sign or air crescent sign. Two (2/44, 4.5%) and 26 (26/44, 59.1%) patients had a positive GM measurement in bronchoalveolar lavage fluid and serum, respectively. About cultures, 56.8% (25/44) of cases were identified as positive for *Aspergillus* spp. (nineteen with *A. fumigatus*, two with *A. flavus* and four with unclassified *Aspergillus*). 

### 3.4. Risk Factors of Mortality in IPA Patients

Among the IPA patients, 17 died within 90 days. There were differences in the outcomes among patients with the complications of respiratory failure, upper gastrointestinal bleeding, and septic shock; the treatments of the ventilator, vasoactive agents and continuous renal replacement therapy, and voriconazole; the symptoms of dyspnea; and the mycological findings of the serum level of GM test (Table 3 and Table 4). The above nine variables were considered for this logistic regression model. Multivariate logistic regression analysis showed that respiratory failure was an independent risk factor for predicting mortality in IPA patients with HBV-ACLF [OR 26, *p* = 0.000] (Table 5).

### 3.5. Treatment with Antifungals

All but four patients (4/44, 9.1%) received antifungal therapy. Twenty-seven (27/44, 61.4%) patients received voriconazole or voriconazole plus as antifungal treatment, eight (18.2%) received caspofungin, and five (11.4%) received micafungin (Table 3).

**Figure 4:**
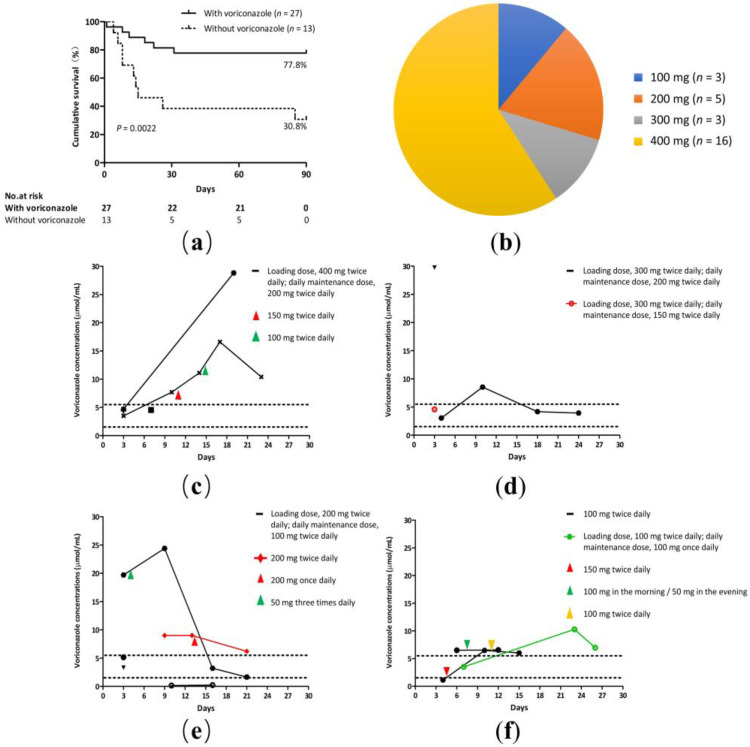
Voriconazole regimen for IPA. (**a**) A cumulative 90-day survival of patients with or without voriconazole regimen. Log-rank test (77.8% vs. 30.8%, *p* = 0.0022). (**b**) Loading doses of voriconazole in HBV-ACLF patients. The loading dose ranged from 100 to 400 mg twice daily. (**c**–**f**) Trough voriconazole concentrations comparing patients treated with different regimens. Nine patients underwent plasma voriconazole concentration monitoring more than twice; the maintenance dose was based on the plasma voriconazole concentration. (**c**) A standard voriconazole regimen. (**d**–**f**) A reduced voriconazole regimen.

Voriconazole is a first-line option for the primary treatment of IPA, and therapeutic regimens consisting of voriconazole therapy were the most common. Patients treated with a voriconazole-based regimen had a higher 90-day survival compared with those treated with a non-voriconazole regimen (77.8% vs. 30.8%, *p* = 0.0022; Figure 4a). However, experience of its use in ACLF patients is limited due to concerns of potential liver injuries. The loading dose ranged from 100 to 400 mg twice daily (Figure 4b). Patients began with the dose of the standard regimen (loading dose, 400 mg twice daily) or reduced regimen (loading dose ranged from 100 to 300 mg twice daily).

Plasma voriconazole concentration measurements were performed as therapeutic drug monitoring in 55.6% (15/27) of the patients. Our hospital’s normal range of plasma voriconazole concentration levels were 1.5 μg/mL to 5.5 μg/mL. Nine patients underwent plasma voriconazole concentration monitoring more than twice; the maintenance dose was based on the plasma voriconazole concentration. The drug concentrations exceeded the safe range in 60% (9/15) of the patients (Figure 4c–f). Out of four patients treated with a standard dose regimen (loading dose, 400 mg twice daily; daily maintenance dose, 200 mg twice daily), in two cases the plasma concentrations exceeded the safe range (Figure 4c). The remaining 11 patients were treated with a reduced dosage regimen. Out of three patients treated with a reduced dose regimen (loading dose, 300 mg twice daily; daily maintenance dose, 150/200 mg twice daily), in two cases the plasma concentrations exceeded the safe range (Figure 4d). Out of four patients treated with a reduced dose regimen (loading dose, 200 mg twice daily; daily maintenance dose, 100 mg twice daily) and one with a reduced dose regimen (200 mg twice daily), in two cases the plasma concentrations exceeded the safe range and in one case the plasma concentration was less than 1.5 μg/mL (Figure 4e). Out of one patient treated with a reduced dose regimen (loading dose, 100 mg twice daily; daily maintenance dose, 100 mg once daily) and two with a reduced dose regimen (100 mg twice daily), all three cases had plasma concentrations that exceeded the safe range (Figure 4f).

## 4. Discussion

This study investigated the prevalence, clinical manifestations, risk factors, outcomes, and antifungal treatment in HBV-ACLF patients with IPA. First, we found the prevalence of IPA in HBV-ACLF patients is 1.84%. Second, we described the detailed clinical features of IPA in HBV-ACLF patients and found that WBC count was the independent factor attributed to the IPA development. Third, we demonstrated that respiratory failure was associated with the mortality of HBV-ACLF patients with IPA. Finally, we suggested that voriconazole should be assessed by closely monitoring blood levels.

A study by W. Wang & C et al. reported that the prevalence of IPA in HBV-ACLF patients is 66/798 (8.3%) [4]. Meanwhile, in the study by Jiajia Chen et al., 37/787 (4.7%) of the patients with HBV-ACLF developed IPA [10]. The short-term mortality observed in these patients ranged from 95% to 100%. In our study, the incidence was 44/2380 (1.84%), and the mortality was 38.6%, which was lower than previous reports. The incidence of IPA is underestimated, and this discrepancy may be attributed to a population selection bias, as our study enrolled patients from only Sichuan Province, a southwest region of China. The IPA rate in patients with HBV-ACLF is not high, but once it occurs, the mortality is very high. W. Wang & C et al. reported that in the context of HBV-ACLF, patients with IPA died mainly due to respiratory failure within a very short time of IPA diagnosis, regardless of various antifungal therapies [4]. Our study’s multivariate logistic regression analysis showed that respiratory failure was a risk factor for predicting mortality in patients with IPA, consistent with previous reports. Therefore, we suggested the importance of a prompt IPA diagnosis to improve HBV-ACLF patients’ outcomes.

Accordingly, a large number of studies have reported lots of IPA risk factors for liver failure to provide methods for early clinical diagnosis and treatment. The previous studies showed age, sex, antibiotics use, steroid exposure, encephalopathy, diabetes, hepatorenal syndrome, frequent invasive procedures, and plasma exchange were independent risk factors associated with the occurrence of IPA in patients with liver failure [4,10,11,12]. In our study, multivariate logistic regression analysis found that WBC count was an independent predictor for IPA. IPA has been traditionally regarded as an infection mainly occurring in patients with well-established risk factors, such as neutropenia. However, an increasing number of reports underline the susceptibility of some categories of nonneutropenic patients to invasive fungal infections [13,14,15]. A study enrolled 43 nonneutropenic patients, and the underlying diseases in these patients included twelve cases of bronchiectasis, eleven cases of old pulmonary tuberculosis, eleven cases of nasosinusitis, nine cases of diabetes, seven cases of chronic obstructive pulmonary disease, three cases of bronchial asthma, two cases of lung cancer, and one case of pulmonary fibrosis [14]. It found that a WBC count > 20.0 × 10^9^/L may aid in the early diagnosis of IPA [14]. Moreover, a case report showed that a diabetes patient with an elevated WBC count (WBC count 33 × 10^9^/L) was diagnosed as having IPA [15]. IPA patients with combined underlying lung disease, diabetes, liver cirrhosis, and so on usually present with nonspecific symptoms and signs as well as nonspecific CT findings, and the diagnosis of IPA in nonneutropenic patients is more challenging. In this study, we, for the first time, confirmed that the high WBC count was a risk factor for IPA in HBV-ACLF patients. This means that patients with HBV-ACLF and a high WBC count will have an increased risk for IPA. Therefore, different WBC counts for diagnosing IPA should be considered based on the underlying condition. The conventional laboratory parameter of WBC count might be used to monitor patients’ progress with HBV-ACLF and IPA.

The effects of antifungal agents and the duration of treatment for IPA in patients with HBV-ACLF remain inadequate. Voriconazole is recommended as the first-line treatment for IPA according to the guidelines of the Infectious Diseases Society of America in 2016 [16]. It is associated with potential liver damage. Voriconazole drug instructions indicate that for patients with mild to moderate liver cirrhosis (Child–Pugh A and B), the loading dose remains unchanged and the maintenance dose is halved. However, no recommendation has been given for patients with severe liver cirrhosis (Child–Pugh C) and liver failure [17]. Jie Gao et al. showed that a voriconazole-based regimen achieved a comparable 90-day survival to ACLF patients with IPA [18]. In agreement with this finding, our analysis showed that patients treated with a voriconazole-based regimen had a higher 90-day survival than those treated with a non-voriconazole regimen (77.8% vs. 30.8%, *p* = 0.0022). How much of the dosage of voriconazole needs to be used in patients with liver failure, given concerns of the hepatotoxicity of voriconazole? To date, there are few studies on the use of voriconazole in ACLF patients, and some researchers have analyzed its individualized regime, but its safety is still unknown. Jie Gao et al. reported that ALCF patients (*n* = 8) treated with an optimal voriconazole regimen (loading doses, 200 mg twice daily; maintenance doses, 100 mg once daily) resulted in rational trough plasma drug concentrations (1–5 μg/mL), 90-day survival rate, and no observed adverse events [18]. Danli Chen et al. enrolled 102 patients treated with a voriconazole regimen; the loading dose of voriconazole ranged from 100 to 800 mg/day, and the maintenance dose ranged from 0 to 800 mg/day [19]. They confirmed that the voriconazole regimen (loading dose, 200 mg twice daily; daily maintenance dose, 100 mg once daily) achieved comparable 28-day survival and optimal trough drug concentrations (1–5 μg/mL) [19]. However, we observed that the drug concentrations exceeded the safe range with a reduced dosage (loading dose ranged from 100 to 300 mg twice daily; maintenance doses, 100 to 200 mg twice daily). This discrepancy may be attributed to factors such as age, liver function, drug interactions, and polymorphisms of human cytochrome P450 enzymes [20,21]. We suggested that voriconazole should be assessed by closely monitoring blood levels and adverse effects; however, as the current investigation included a small number of patients for therapeutic drug monitoring, a large sample, prospective study is warranted to validate the optimal voriconazole regimen for IPA in HBV-ACLF patients.

*A. fumigatus* is the primary causative agent of IPA. In our study, the *A. fumigatus* species remained the most commonly identified species, with a proportion of 43.2% of tested clinical isolates. However, studies have also observed that isolates of cryptic *Aspergillus* species and other sections can cause IPA [22]. Cryptic *Aspergillus* species are morphologically indistinguishable from others but differentiated by molecular methods. They often show intrinsic resistance to several classes of antifungals, making some treatments less effective. Furthermore, the diagnostic challenges of accurately identifying cryptic *Aspergillus* species, frequently misidentified as *A. fumigatus*, can lead to the inappropriate use of antifungals. Therefore, we should improve the awareness of the importance of cryptic *Aspergillus* species in the clinical setting. Recently, voriconazole resistance has been an increasing problem in invasive aspergillosis. A multicenter retrospective cohort study by Pieter P Lestrade et al. found that 37/196 (19%) cases of invasive aspergillosis in patients with *A. fumigatus* positive cultures harbored a voriconazole-resistant infection [23]. We should also be alert to the possible occurrence of voriconazole resistance.

Our study had some limitations. First, the study was a retrospective design. Second, this was a single-center study, and the generalizability of the results may be limited. Third, lung biopsies could not be performed in patients due to their coagulation function and platelet status, which will not lead to a precise diagnosis.

## 5. Conclusions

In summary, IPA is more common in HBV-ACLF patients with high WBC counts. Once IPA occurs in patients with HBV-ACLF, the fatality rate is very high. Respiratory failure was associated with the mortality of HBV-ACLF patients with IPA. A voriconazole regimen is needed for such critical patients, and therapeutic drug monitoring should be performed. Clinicians should increase the awareness of the risk factors of IPA in patients with HBV-ACLF and make early diagnoses and treatments to improve patients’ prognoses.

## Figures and Tables

**Table 1:** Characteristics of patients with HBV-ACLF in the IPA and control groups.

Characteristics	All Patients with HBV-ACLF (*n* = 132)	Control (*n* = 88)	IPA (*n* = 44)	*p* Value
Age (years)	48.00 (40.25, 57.00)	47.00 (41.00, 53.00)	50.00 (37.50, 59.75)	0.277
Sex (male)	120 (90%)	78 (88.6%)	42 (95.5%)	0.199
Hospital days	24.50 (15.00, 39.00)	27.50 (17.00, 39.00)	22.00 (9.50, 38.75)	0.072
Underlying disease				
Cardiovascular disease	14 (10.6%)	2 (2.3%)	12 (27.3%)	0.000 *
Diabetes mellitus type 2	8 (6.1%)	2 (2.3%)	6 (13.6%)	0.017 *
Liver cirrhosis	103 (78%)	72 (81.8%)	31 (70.5%)	0.137
Complications				
Hepatic encephalopathy	33 (25%)	18 (20.5%)	15 (34.1%)	0.088
Hepatorenal syndrome	31 (23.5%)	22 (25.0%)	9 (20.5%)	0.561
Spontaneous bacterial peritonitis	89 (67.4%)	61 (69.3%)	28 (63.6%)	0.511
Upper gastrointestinal bleeding	23 (17.4%)	8 (9.1%)	15 (34.1%)	0.000 *
Septic shock	7 (5.3%)	0	7 (15.9%)	0.000 *
Laboratory findings				
Log HBV DNA (IU/mL)	4.32 (3.05, 5.62)	4.60 (3.00, 5.72)	4.25 (3.05, 5.44)	0.338
Total bilirubin (μmol/L)	380.45 (307.22, 455.40)	379.40 (308.42, 454.50)	386.50 (300.35, 458.28)	0.813
Alanine aminotransferase (IU/L)	129.00 (70.25, 273.50)	160.00 (84.25, 342.75)	95.00 (51.25, 171.75)	0.001 *
Aspartate aminotransferase (IU/L)	144.50 (83.00, 226.50)	176.50 (114.00, 262.75)	91.00 (70.00, 145.00)	0.000 *
Albumin (g/L)	31.25 (29.13, 34.10)	31.50 (29.78, 34.22)	30.50 (27.90, 34.10)	0.262
Globulin (g/L)	25.80 (20.90, 31.20)	28.40 (23.55, 33.65)	20.90 (15.00, 25.30)	0.000 *
Creatinine (μmol/L	80.50 (66.25, 103.00)	80.50 (67.25, 105.25)	80.50 (61.25, 101.50)	0.516
Serum sodium (mmol/L)	135.90 (132.40, 138.30)	136.00 (133.00, 138.60)	134.60 (129.70, 138.30)	0.236
Ammonia (μmol/L)	69.00 (48.25, 97.75)	69.50 (49.75, 97.00)	67.35 (44.90, 110.35)	0.892
White blood cell count (×10^9^/L)	7.05 (5.15, 10.28)	6.59 (4.77, 8.27)	9.97 (6.55, 16.51)	0.000 *
Neutrophil ratio (%)	73.80 (65.00, 81.93)	71.10 (62.95, 76.95)	82.55 (71.80, 88.63)	0.000 *
Hemoglobin (g/L)	127.00 (113.00, 136.00)	129.00 (117.50, 136.75)	123.00 (93.00, 134.00)	0.008 *
Platelet count (×10^9^/L)	84.50 (55.50, 117.00)	93.50 (66.00, 124.75)	61.00 (36.00, 101.00)	0.001 *
International normalized ratio	2.01 (1.70, 2.45)	2.01 (1.69, 2.48)	2.00 (1.70, 2.41)	0.965
Procalcitonin (ng/mL)	0.74 (0.46, 1.39)	0.66(0.43, 0.94)	1.23 (0.59, 2.39)	0.000 *
MELD	24.98 (22.33, 29.09)	25.07 (22.74, 28.81)	24.97 (21.45, 29.76)	0.678
MELD-Na	27.16 (23.72, 31.01)	26.83 (23.93, 30.21)	27.43 (23.42, 31.72)	0.877
Antibiotic usage	101 (76.5%)	57 (64.8%)	44 (100%)	0.000 *
Steroid or immunosuppressant exposure	8 (6.1%)	0	8 (18.2%)	0.000 *
Artificial liver support system	80 (60.6%)	49 (55.7%)	31 (70.5%)	0.102
Outcome (dead)	31 (23.5%)	14 (15.9%)	17 (38.6%)	0.004 *

HBV DNA, hepatitis B virus deoxyribonucleic acid; MELD, model for end-stage liver disease; MELD-Na, MELD-sodium. The χ^2^ test or Fisher’s exact probability test was used to examine categorical variables. The Mann–Whitney U test was used to analyze continuous variables. * Values were statistically significant at *p* < 0.05.

**Table 2:** Logistic regression analysis of risk factors associated with IPA among patients with HBV-ACLF.

Factor	Odds Ratio	95%CI		*p* Value
		Lower	Upper	
White blood cell count	1.468	1.045	2.062	0.027 *

CI, confidence interval. Predictors for the development of IPA were calculated using multivariable binomial logistic regression analysis. * Values were statistically significant at *p* < 0.05.

**Table 3:** The characteristics of HBV-ACLF patients with IPA.

Characteristics	IPA (*n* = 44)	Recovery (*n* = 27)	Death (*n* = 17)	*p* Value
Age (years)	50.00 (37.50, 59.75)	48.00 (37.00, 57.00)	57.00 (44.50, 63.50)	0.111
Sex (male)	42 (95.5%)	25 (92.6%)	17 (100%)	0.515
Smoking history	23 (52.3%)	15 (55.6%)	8 (47.1%)	0.758
Underlying disease				
Cardiovascular disease	12 (27.3%)	5 (18.5%)	7 (41.2%)	0.164
Diabetes mellitus type 2	6 (13.6%)	3 (11.1%)	3 (17.6%)	0.662
Liver cirrhosis	31 (70.5%)	20 (74.1%)	11 (64.7%)	0.521
Complications				
Respiratory failure	16 (36.4%)	3 (11.1%)	13 (76.5%)	0.000 *
Hepatic encephalopathy	15 (34.1%)	6 (22.2%)	9 (52.9%)	0.053
Hepatorenal syndrome	9 (20.5%)	3 (11.1%)	6 (35.3%)	0.068
Spontaneous bacterial peritonitis	28 (63.6%)	19 (70.4%)	9 (52.9%)	0.337
Upper gastrointestinal bleeding	15 (34.1%)	5 (18.5%)	10 (58.8%)	0.009 *
Septic shock	7 (15.9%)	0	7 (41.2%)	0.001 *
Comprehensive treatment measure				
Transferred to intensive care	5 (11.4%)	1 (3.7%)	4 (23.5%)	0.065
Ventilator	13 (29.5%)	1 (3.7%)	12 (70.6%)	0.000 *
Artificial liver support system	31 (70.5%)	19 (70.4%)	12 (70.6%)	1.000
Liver transplantation	4 (9.1%)	2 (7.4%)	2 (11.8%)	0.634
Vasoactive agents	7 (15.9%)	1 (3.7%)	6 (35.3%)	0.009 *
Continuous renal replacement therapy	6 (13.6%)	1 (3.7%)	5 (29.4%)	0.025 *
Steroid or immunosuppressant exposure	10 (22.7%)	4 (14.8%)	6 (35.3%)	0.150
Antibiotic usage				
One antibiotic	9 (20.5%)	8 (29.6%)	1 (5.9%)	0.052
Two antibiotics	24 (54.5%)	11 (40.7%)	13 (76.5%)	
Three or more antibiotics	11 (25%)	8 (29.6%)	3 (17.6%)	
Antibiotic categories				
Carbapenems	27 (61.4%)	13 (48.1%)	14 (82.4%)	
Beta-lactamase inhibitors	29 (65.9%)	19 (70.4%)	10 (58.8%)	
Cephalosporins	12 (27.3%)	10 (37.0%)	2 (11.8%)	
Glycopeptides	14 (31.8%)	9 (33.3%)	5 (29.4%)	
Tetracyclines	2 (4.5%)	0	2 (11.8%)	
Quinolones	7 (15.9%)	6 (22.2%)	1 (5.9%)	
Polymyxins	3 (6.8%)	0	3 (17.6%)	
Antifungal treatment				
Voriconazole	20 (45.5%)	16 (59.3%)	4 (23.5%)	0.030 *
Voriconazole plus	7 (15.9%)	5 (18.5%)	2 (11.8%)	0.689
Caspofungin	8 (18.2%)	3 (11.1%)	5 (29.4%)	0.227
Micafungin	5 (11.4%)	1 (3.7%)	4 (23.5%)	0.065
Untreated	4 (9.10%)	2 (7.4%)	2 (11.8%)	0.634

The χ^2^ test or Fisher’s exact probability test was used to examine categorical variables. The Mann–Whitney U test was used to analyze continuous variables. * Values were statistically significant at *p* < 0.05.

**Table 4:** Clinical manifestations, imaging findings, and laboratory parameters of HBV-ACLF patients with IPA.

Characteristics	IPA (*n* = 44)	Recovery (*n* = 27)	Death (*n* = 17)	*p* Value
Signs or symptoms				
Fever	17 (38.6%)	7 (25.9%)	10 (58.8%)	0.055
Cough	16 (36.4%)	11 (40.7%)	5 (29.4%)	0.531
Dyspnea	19 (43.2%)	5 (18.5%)	14 (82.4%)	0.000 *
Hemoptysis	5 (11.4%)	2 (7.4%)	3 (17.6%)	0.359
Imaging findings				
Patchy shadow	25 (6.8%)	14 (51.9%)	11 (64.7%)	0.535
Nodules	31 (70.5%)	20 (74.1%)	11 (64.7%)	0.521
Consolidation	19 (43.2%)	10 (37%)	9 (52.9%)	0.359
Halo sign	1 (2.3%)	0	1 (5.9%)	0.386
Cavitary lesion	10 (22.7%)	9 (33.3%)	1 (5.9%)	0.062
Air crescent sign	1 (2.3%)	0	1 (5.9%)	0.386
Pleural effusion	5 (11.4%)	3 (11.1%)	2 (11.8%)	1.000
Mycological findings				
GM test	28	17	11	
BALF cases	2 (4.5%)	1 (3.7%)	1 (5.8%)	
BALF level	1.085	1	1.17	
Serum cases	26 (59.1%)	16 (63%)	10 (58.8%)	
Serum level	1.14 (0.73, 4.63)	0.91 (0.62, 1.35)	5.22 (1.63, 7.62)	0.003 *
Sputum culture	25 (56.8%)	17 (62.9%)	8 (47.1%)	0.359
*Aspergillus fumigatus*	19 (43.2%)	13 (48.1%)	6 (35.3%)	0.535
*Aspergillus flavus*	2 (4.5%)	2 (7.4%)	0	0.515
Unclassified *Aspergillus*	4 (9.1%)	2 (7.4%)	2 (11.8%)	0.634
Laboratory findings				
Log HBV DNA (IU/mL)	4.25 (3.05, 5.44)	3.94 (3.14, 5.08)	4.40 (3.05, 5.86)	0.792
Total bilirubin (μmol/L)	386.50 (300.35, 458.28)	369.40 (254.80, 455.50)	392.90 (334.25, 499.85)	0.252
Alanine aminotransferase (IU/L)	95.00 (51.25, 171.75)	96.00 (57.00, 178.00)	76.00 (45.00, 155.50)	0.563
Aspartate aminotransferase (IU/L)	91.00 (70.00, 145.00)	82.00 (73.00, 175.00)	94.00 (62.50, 139.00)	0.914
Albumin (g/L)	30.50 (27.90, 34.10)	29.70 (27.60, 34.10)	30.60 (29.60, 34.70)	0.341
Globulin (g/L)	20.90 (15.00, 25.30)	20.55 (17.43, 25.90)	20.90 (14.10, 24.45)	0.700
Creatinine (μmol/L)	80.50 (61.25, 101.50)	80.00 (59.00, 97.00)	82.00 (67.50, 103.00)	0.579
Serum sodium (mmol/L)	134.60 (129.70, 138.30)	134.10 (130.00, 138.10)	136.40 (128.90, 139.90)	0.306
Ammonia (μmol/L)	67.35 (44.90, 110.35)	66.00 (49.50, 97.00)	73.40 (41.10, 119.00)	0.700
White blood cell count (×10^9^/L)	9.97 (6.55, 16.51)	10.43 (7.20, 16.74)	9.68 (4.46, 14.47)	0.479
Neutrophil ratio (%)	82.55 (71.80, 88.63)	83.05 (70.23, 88.10)	82.15 (76.73, 88.68)	0.604
Hemoglobin (g/L)	123.00 (93.00, 134.00)	123.00 (96.00, 134.00)	106.50 (84.75, 134.75)	0.269
Platelet count (×10^9^/L)	61.00 (36.00, 101.00)	54.00 (34.50, 101.75)	67.00 (39.00, 102.00)	0.623
International normalized ratio	2.00 (1.70, 2.41)	1.96 (1.70, 2.32)	2.34 (1.70, 2.79)	0.185
Procalcitonin (ng/mL)	1.23 (0.59, 2.39)	1.24 (0.55, 2.42)	1.23 (0.62, 2.47)	0.980
MELD score	24.97 (21.45, 29.76)	24.1424 (20.85, 28.67)	26.32 (23.09, 31.98)	0.159
MELD-Na score	27.43 (23.42, 31.72)	26.02 (23.34, 31.93)	28.21 (24.50, 32.43)	0.539

GM, galactomannan; BALF, bronchoalveolar lavage fluid; HBV DNA, hepatitis B virus deoxyribonucleic acid; MELD, model for end-stage liver disease; MELD-Na, MELD-sodium. The χ^2^ test or Fisher’s exact probability test was used to examine categorical variables. The Mann–Whitney U test was used to analyze continuous variables. * Values were statistically significant at *p* < 0.05.

**Table 5:** Logistic regression analysis of risk factors associated with mortality in HBV-ACLF patients with IPA.

Factor	Odds Ratio	95% CI		*p* Value
		Lower	Upper	
Respiratory failure	26	5.033	134.313	0.000 *

CI, confidence interval. Predictors for the development of IPA were calculated using multivariable binomial logistic regression analysis. Survival analysis was presented using the Kaplan–Meier method and compared by the log-rank test. * Values were statistically significant at *p* < 0.05.

## Data Availability

Data associated with this study has not been deposited into a publicly available repository and will be made available from the corresponding author on reasonable request.
